# A Network Pharmacology Approach to Reveal the Underlying Mechanisms of *Artemisia annua* on the Treatment of Hepatocellular Carcinoma

**DOI:** 10.1155/2021/8947304

**Published:** 2021-02-22

**Authors:** Shuqiao Zhang, Zhuomao Mo, Shijun Zhang, Xinyu Li

**Affiliations:** ^1^Hospital of Chengdu University of Traditional Chinese Medicine, Chengdu University of Traditional Chinese Medicine, Chengdu, Sichuan 611137, China; ^2^Department of Traditional Chinese Medicine, The First Affiliated Hospital, Sun Yat-Sen University, Guangzhou, Guangdong 510080, China; ^3^Clinical Medical College of Acupuncture Moxibustion and Rehabilitation, Guangzhou University of Traditional Chinese Medicine, Guangzhou, Guangdong 510080, China

## Abstract

**Objective:**

To investigate the potential active ingredients and underlying mechanisms of *Artemisia annua* (AA) on the treatment of hepatocellular carcinoma (HCC) based on network pharmacology.

**Methods:**

In the present study, we used a network pharmacological method to predict its underlying complex mechanism of treating HCC. First, we obtained relative compounds of AA based on the traditional Chinese medicine systems pharmacology (TCMSP) database and collected potential targets of these compounds by target fishing. Then, we built HCC-related targets target by the oncogenomic database of hepatocellular carcinoma (OncoDB.HCC) and biopharmacological network (PharmDB-K) database. Based on the matching results between AA potential targets and HCC targets, we built a protein-protein interaction (PPI) network to analyze the interactions among these targets and screen the hub targets by topology. Furthermore, the function annotation and signaling pathways of key targets were performed by Gene Oncology (GO) and Kyoto Encyclopedia of Genes and Genomes (KEGG) enrichment analysis using DAVID tools. Finally, the binding capacity between active ingredients and key targets was validated by molecular docking.

**Results:**

A total of 19 main active ingredients of AA were screened as target prediction; then, 25 HCC-related common targets were seeked out via multiple HCC databases. The areas of nodes and corresponding degree values of EGFR, ESR1, CCND1, MYC, EGF, and PTGS2 were larger and could be easily found in the PPI network. Furthermore, GO and KEGG enrichment analysis showed that these key targets were significantly involved in multiple biological processes and pathways which participated in tumor cell proliferation, apoptosis, angiogenesis, tumor invasion, and metastasis to accomplish the anti-HCC activity. The molecular docking analysis showed that quercetin could stably bind to the active pocket of EGFR protein 4RJ5 via LibDock.

**Conclusion:**

The anticancer effects of AA on HCC were predicted to be associated with regulating tumor cell proliferation, apoptosis, angiogenesis, tumor invasion, and metastasis via various pathways such as the EGFR signaling pathway, ESR1 signaling pathway, and CCND1 signaling pathway. It is suggested that AA might be developed as a broad-spectrum antitumor drug based on its characteristics of multicomponent, multipath, and multitarget.

## 1. Introduction

Hepatocellular carcinoma (HCC) is the second most lethal cancer worldwide with persistently increasing mortality in the Asia-Pacific region [1, 2]. HCC is highly refractory to therapeutic interventions. Even after surgical resection or ablation, 70% of patients experience tumor recurrence within 5 years. Thus, HCC represents a major public health problem in the Asia-Pacific region [2, 3].

Traditional Chinese medicines, unique biomedical and pharmaceutical resources, have been widely used in the prevention and treatment HCC [4, 5]. *Artemisia annua* (AA) is a Chinese medicinal plant, which is used throughout Asia and Africa as tea or press juice to treat malaria. Since the 2015 Nobel Prize in Physiology or Medicine conferred to Chinese scientist, Youyou Tu, AA drew attention worldwide [6]. AA was found to have anticancer activity in vitro and in vivo [3–6]. AA is not only widely used in the treatment of various tumors but also in the prevention of tumors [6, 7]. AA also exerts synergistic antitumor effects with a wide array of clinically established drugs [8–10]. Because AA has many kinds of active ingredients, the target and mechanism of its anti-HCC effect have not been completely clear.

Network pharmacology combines system biology, bioinformatics, multidirectional pharmacology, and other disciplines organically, emphasizing the synergistic effect of drug components on complex diseases [11]. It can systematically clarify the mechanism of traditional Chinese medicine by showing the relationship among drugs, targets, and diseases, providing new ideas for the research and development of traditional Chinese medicine and new drugs [12]. Therefore, this study systematically studies the mechanism and material basis of AA in the treatment of HCC by using the method of network pharmacology, which will provide a new theoretical basis and direction for the systematic development of AA.

## 2. Materials and Methods

### 2.1. Database and Software

The databases involved in this study are OncoDB.HCC (genomics database of HCC, http://oncodb.hcc.ibms.sinica.edu.tw/), PharmDB-K (http://pharmdb.org/), PubChem (database of biological activity of organic small molecules, http://pubchem.ncbi.nlm.nih.gov), Protein Data Bank (PDB, https://www.rcsb.org/), STRING (https://string-DB.Org/), TCMSP (pharmacology database and analysis platform of the traditional Chinese medicine system, http://lsp.nwu.edu.cn/tcmsp.php), and UniProt (https://www.uniprot.org). The data analysis software includes Cytoscape 3.7.1 (plug-ins ClueGO 2.5.1 and CluePedia 1.5.1), Discovery Studio, and Venn diagrams (OmicShare tools).

### 2.2. Identification of Chemical Ingredients in AA

All the active components of AA were collected from TCMSP platform, and the screening conditions were based on drug-likeness (DL) ≥0.18 and oral bioavailability (OB) ≥30%. Enter the active ingredients into PubChem to search for the corresponding molecular structure and record the corresponding PubChem CID. Then, based on the TCMSP database, the potential targets of the active ingredients were matched one by one, and then, the target protein and gene information were corrected through the UniProt database to select the target of “*Homo sapiens*,” which is the prediction target of the main active ingredients of AA.

### 2.3. Prediction of Compound-Related Targets

With “hepatocellular carcinoma” as the keyword, OncoDB.HCC (http://oncodb.hcc.ibms.sinica.edu.tw/index.htm) and PharmDB-K (http://pharmdb.org) were used to search and screen the known disease-targets for the subsequent study, and the repeated targets in the search results were discarded. UniProtKB (http://www.uniprot.org/) was used to get the standard targets' names with the organism selected as “*Homo sapiens*.” Then, the predicted targets of *Artemisia annua* major active components and HCC-related targets were intersected, and the Venn plot was drawn to extract common target genes.

### 2.4. Identification of HCC-Related Targets

**Figure 1:**
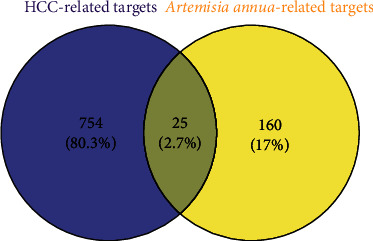
Proteins interactions network of *Artemisia annua*.

The common targets of drug and disease were found, and a Venn diagram was drawn (Figure 1). Next, the common targets were inputted into STRING (http://string-db.org/, version 10.5) for PPI analysis, and Cytoscape 3.6.1 (http://www.cytoscape.org/) was adopted to visualize the results. The STRING database refers to a biological database for prediction of PPIs, with data from several sources (e.g., experimental data, computational prediction methods, and a public text set). Next, the protein interaction relationship was obtained, and the results were exported in the tab-separated value (TSV) format. STRING integrates known and estimated PPIs and then assesses PPI with confidence score ranges (low confidence: <0.4; medium: 0.4–0.7; high: >0.7; and highest: >0.9). In this study, PPIs with medium confidence scores (>0.4) were gained for further exploration. The topological analysis covers degree centrality (DC), betweenness centrality (BC), closeness centrality (CC), eigenvector centrality (EC), network centrality (NC), and local average connectivity (LAC). DC is a vital parameter to measure the local centrality of a node. A node with high DC score can be the central node of the network. It often engages in essential life activities, executes a key biological function, and plays an irreplaceable role to maintain the stability of the interaction network, making it a research hotspot for drug targets. BC refers to the number of times a node acts as the shortest bridge between the other two nodes. The higher a node mediates, the more centrality it has. In this experiment, the nodes with DC score and BC score greater than 2 times the median were taken as important nodes in the network, namely, “key targets.” We assumed that they were the critical targets for antidiabetes in astragalus. In this study, the median of DC was 4, and the median of BC was 0.00558038.

### 2.5. GO and KEGG Pathway Enrichment Analysis

Using the plugins of ClueGO and CluePedia of Cytoscape software, take *P* < 0.05 as the screening condition of statistical difference. GO analysis is a system extensively used for gene function classification in the field of biology, which is primarily adopted to express the function of gene products, including cell function, molecular function (MF), and biological process (BP). Then, the biological function and pathway analysis of AA's potential HCC target were carried out. Finally, the network topology of AA active component target gene signal pathway was constructed.

### 2.6. Validation of the Binding Capacity between Active Ingredients and Key Targets by Molecular Docking

Based on the results of the above steps, the active component with the largest number of targets was selected as the ligand, and the target with the largest “degree” in the PPI network was selected as the receptor for molecular docking. The three-dimensional structure of the active ingredient in PubChem CID and the protein structure of the target in PDB database were imported to Discovery Studio software for routine molecular structure preprocessing according to the method of molecular docking [13]; then, the LibDock docking mode was selected, and the docking parameters were the default.

## 3. Result

### 3.1. Active Components and Target Selection of AA

After screening, a total of 19 main components were included in the study, target prediction was carried out for these 19 components, and 510 targets were extracted after deduplication as shown in Table 1.

### 3.2. Screening of HCC Targets

**Figure 2:**
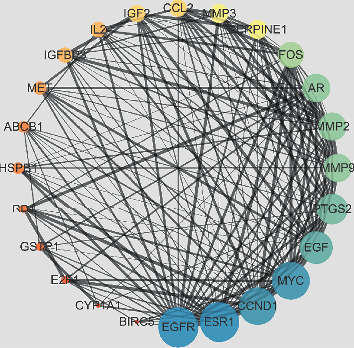
Screening of antihepatocarcinoma targets of *Artemisia annua*.

The databases OncoDB.HCC and PharmDB-K were used to retrieve 613 and 309 HCC-related targets, respectively, and then, the human gene name transformation was carried out through UniProt database. And then, a total of 779 hepatoma-related target genes were obtained after further gene deduplication. The abovementioned hepatoma-related target genes and 185 predicted target genes were input into Venn diagrams software for intersection and construction of Venn diagrams as shown in Figure 2. 25 common target genes, namely, AA potential anti-HCC targets, were obtained as shown in Table 2.

### 3.3. PPI Network Construction of Liver Cancer Target

As shown in Figure 1, the above 25 target genes were uploaded to the STRING platform, and the species was set as “*Homo sapiens*,” and the rest parameters were defaulted to obtain the protein interaction data information; then, they were imported into the software of Cytoscape to draw the PPI network. In the PPI network, 25 nodes (target genes) interact through 161 edges. The size and color depth of the node are set according to the degree, that is, the larger the node is, the greater the degree of the target gene corresponding to the darker the color is, and the score of the relationship value between the two target genes is expressed by the thickness of the edge, that is, the higher the combination score, the thicker the line of the edge.

### 3.4. Biological Function and Signal Pathway Analysis

**Figure 3:**
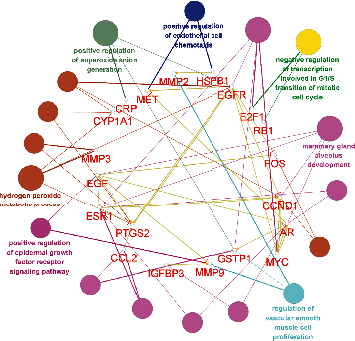
Biological function analysis of potential antihepatocarcinoma targets from main active ingredients of *Artemisia annua*.

Go enrichment analysis was used to analyze the biological process of AA potential antihepatoma target. The results showed that genes such as EGFR, ESR1, CCND1, MYC, EGF, and PTGS2 were involved in two or more pathways as cross-talk genes as shown in Figure 3, and the specific information of signal pathways are shown in Table 3.

### 3.5. Network Diagram of *Artemisia annua* Active Component Liver Cancer Target Gene Pathway

**Figure 4:**
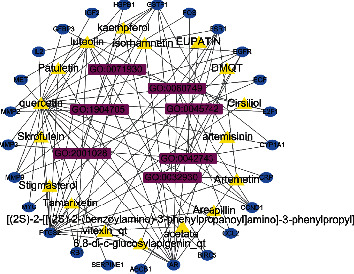
Network of ingredients-targets-signal pathways of *Artemisia annua*.

Based on the software platform of Cytoscape, the network diagram of the GO signal pathway of AA's main active ingredients treating liver cancer target gene is constructed with merge plug-in, including 17 nodes of AA's main active ingredients, 25 nodes of target gene, and 7 nodes of the GO signal pathway. Node and node edge represent the interaction between the active components, liver cancer target genes, and pathways as shown in Figure 4.

### 3.6. The Binding Capacity between Active Compounds and Key Targets by Molecular Docking

**Figure 5:**
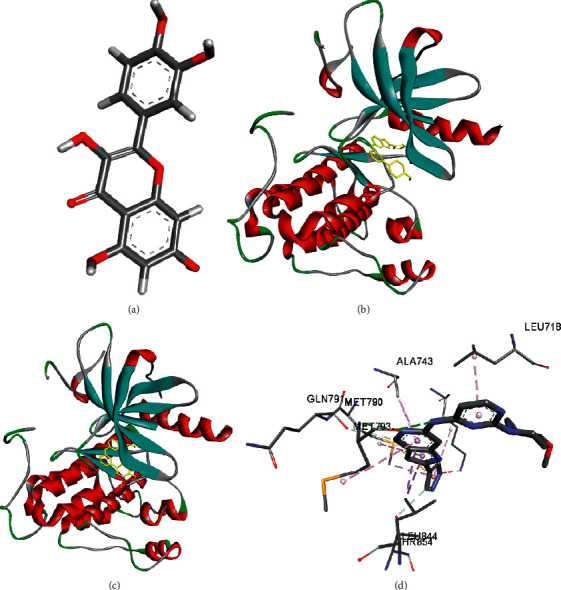
Diagrams of interaction of quercetin with EGFR (4RJ5).

According to the above results, quercetin is the most active component to predict the target, and EGFR is the most important target in the PPI network. Therefore, they are selected as the ligands and receptors for molecular docking. The CID of quercetin was 5280343 on PubChem, and its three-dimensional structure was downloaded, as shown in Figure 5(a). The EGFR protein ID was 4rj5 on PDB, and its structure was downloaded (Figure 5(b)). The results showed that quercetin could stably bind to the active pocket of EGFR protein 4RJ5 (Figure 5(c)) via LibDock; they had H-bond via amino acid residue MET793; pi bond via LEU844, MET790, ALA743, and LEU718; and C-H bond via GLN791 and THR854.

The yellow structure of EGFR (4JR5) protein shown in Figures 5(b) and 5(c) is its active pocket.

## 4. Discussion

In traditional Chinese medicine (TCM) theory, heat-clearing and detoxicating (HCD) herbs are essential components of TCM formulas for cancer treatment [14]. Accumulated evidences have shown that HCD herbs exhibited remarkable anticancer effects when used alone or combined with other therapeutic approaches, and the extracts or pure compounds of the HCD herbs showed a broad anticancer spectrum without significant toxic effects [15, 16]. As an important HCD herb, AA's antitumor effect has attracted more and more attention [17, 18].

In the current study, there were 17 main active ingredients including luteolin, quercetin, isorhamnetin, kaempferol, stigmasterol, eupatin, tamarixetin, patuletin, areapillin, artemetin, skrofulein, cirsiliol, vitexin-qt, DMQT, [(2S)-2-[[(2S)-2-(benzoylamino)-3-phenylpropanoyl]amino]-3-phenylpropyl] acetate, 6,8-di-c-glucosylapigenin-qt, and artemisinin identified as the potential active ingredients of AA, of which the biological activities against HCC were reported previously. For example, several studies reported antiproliferative and proapoptotic effects of quercetin in HCC, and the beneficial activities of quercetin were also demonstrated in hepatoprotective effects with antioxidant and anti-inflammatory properties [19, 20]. Dihydroartemisinin (DHA), a semisynthetic derivative of artemisinin, could significantly inhibit HCC cell growth in vitro and in vivo via inducing G2/M cell cycle arrest, apoptosis, and excessive intracellular ROS generation in cancer cells; the intraperitoneal injection of DHA resulted in significant inhibition of HCC-xenograft tumors. In a HCC-xenograft mouse model [21, 22], luteolin was demonstrated to exhibit anti-HCC activities, including the induction of apoptosis, cell cycle arrest, and antiangiogenesis; the combination of luteolin and sorafenib could synergistically kill HCC cells through JNK-mediated apoptosis [23, 24]. Therefore, the above main active ingredients indicate the effectiveness and diversity of chemical ingredients in AA for treating HCC.

The results of AA potential target-HCC target network analysis acquired 25 key targets of AA acting on HCC. These key targets were mainly involved in biological processes such as tumor cell proliferation, apoptosis, cell cycle regulation, epithelial stromal transformation, angiogenesis, tumor invasion and metastasis, tumor signal transduction, immune regulation, drug resistance, and other important biological processes [25–28]. The predicted results in our current study were consistent with some previous publications. It was reported that quercetin could not only inhibit the proliferation of HCC cells that relied on aerobic glycolysis but also suppress the progression of HCC by decreasing the protein levels of HK2 and suppressing the AKT/mTOR pathway in HCC cells [29]. In addition, quercetin could enhance ZD55-TRAIL-mediated growth inhibition and apoptosis in HCC cells in vitro experiments, and combined quercetin and ZD55-TRAIL treatment resulted in significantly greater reduction in tumor growth and volume in vivo experiments in mice injected with HuH-7 cells [20].

As observed in the results of KEGG pathway enrichment analysis, the key targets of AA acting on HCC were mainly related to the EGFR signaling pathway, ESR1 signaling pathway, CCND1 signaling pathway, MYC signaling pathway, PTGS2 signaling pathway, and so on. HCC is a heterogeneous disease that the underlying mechanisms have not been clearly elucidated. It is recognized that the pathogenesis of HCC is associated with various biological processes such as tumor cell proliferation, apoptosis, cell cycle regulation, epithelial stromal transformation, angiogenesis, and tumor signal transduction. It was reported that the EGF-EGFR pathway was involved in the development of inflammatory microenvironment in HCC; EGF might facilitate DNA synthesis, regeneration, tumor growth, and progression of HCC cells and bind with EGFR as the potential connection between inflammation and HCC [28]. MYC was found to be a master oncogenic driver, regulating transcriptional programs to influence cell proliferation and metabolism of HCC. MYC amplification co-occurring with EGFR activation was frequently observed in advanced HCC [27]. In addition, the crucial proteins of the PTGS2 signaling pathway such as AKT, STK33, and MTOR were all elevated in COX-2-driven HCC; thus, targeting these proteins could active these oncogenic cascades by COX-2 overexpression during the pathogenesis of HCC [30]. More importantly, these pathways and genes might cooperate in promoting metastasis of HCC through crosstalk between cancer and immune microenvironment [27–29].

Molecular docking is a useful method of predicting binding strength between TCM ingredients and targets based on the spatial structure of ligands and receptors [31]. The molecular docking assay in this study showed that quercetin were conferred strong binding activity with EGFR and further demonstrated that EGFR was the key target related to the treatment of AA for HCC. EGFR pathway, the important signaling of multiple pathways, participated in angiogenesis regulation, immune evasion, apoptosis inhibition, cell proliferation, tumorigenesis, progression, and metastasis of HCC [28, 30, 32]. Several studies demonstrated that the downstream PI3K/Akt, ERK, and JNK signaling pathways of EGFR were activated in cancer metastasis of HCC, and the inhibition of EGFR or its downstream signal pathway would inhibit cancer metastasis [28, 30, 33]. The silencing of EGFR by miR-302b or siEGFR led to downregulation of proliferation-related proteins, such as AKT2, CCND1, and CDK2, and resulted in the inhibition of proliferation in hepatocellular carcinoma SMMC-7721 cells [34]. In addition, the inhibitory effects of afatinib on EMT and tumorigenesis might be associated with the ERK-VEGF/MMP9 signaling pathway, which indicated that targeting EGFR signaling pathway-related proteins was a promising strategy for AA in the treatment of HCC [32]. Quercetin was reported to suppress tumor cell proliferation, cellular protein phosphorylation, and matrix metalloproteinase secretion via the EGFR signaling pathway and further demonstrated that EGFR was the key target related to the treatment of quercetin for HCC, which was consistent with the result of molecular docking in our current study [35, 36].

It is usually difficult to reveal the effect of the active ingredients of AA on the targets and its activity of treating HCC alone because of the complex ingredients of AA and limited experimental research methods at present. However, this study showed that multiple targets and pathways might involve in the occurrence of HCC, and the possible molecular mechanism of AA active ingredients was revealed for the first time based on the combined methods of network pharmacology and molecular docking technology, which indicated that it might be an effective method to reveal the holistic characteristics of AA in the treatment of HCC by using combined methods of network pharmacology and molecular docking technology. Furthermore, quercetin has many targets, which may lead to off-target effects. But we only focused on the key targets that linked the active compounds and HCC. And the therapeutic effect of TCM not only depends on the unique target.

## 5. Conclusions

In conclusion, our study predicted the targets of the ingredients of the AA and explored the underlying mechanism of the potential anti-HCC effects via the method of network pharmacology. The anticancer effects of AA on HCC were predicted to be associated with regulating tumor cell proliferation, apoptosis, angiogenesis, tumor invasion, and metastasis via various pathways such as EGFR signaling pathway, ESR1 signaling pathway, and CCND1 signaling pathway. It is suggested that AA might be developed as a broad-spectrum antitumor drug based on its characteristics of multicomponent, multipath, and multitarget and has a broad application prospect.

## Figures and Tables

**Table 1:** Main active ingredients of *Artemisia annua*.

TCMSP ID	PubChem CID	Compound	Target number
MOL000006	5280445	Luteolin	57
MOL000098	5280343	Quercetin	154
MOL000354	5281654	Isorhamnetin	37
MOL000359	12303645	Sitosterol	3
MOL000422	5280863	Kaempferol	63
MOL000449	5280794	Stigmasterol	31
MOL002235	5317287	Eupatin	16
MOL004083	5281699	Tamarixetin	15
MOL004112	5281678	Patuletin	11
MOL004609	158311	Areapillin	17
MOL005229	5320351	Artemetin	23
MOL007274	188323	Skrofulein	11
MOL007401	160237	Cirsiliol	10
MOL007404	N/A	Vitexin_qt	15
MOL007412	5281603	DMQT	10
MOL007415	10026486	[(2S)-2-[[(2S)-2-(Benzoylamino)-3-phenylpropanoyl]amino]-3-phenylpropyl] acetate	5
MOL007423	N/A	6,8-Di-c-glucosylapigenin_qt	16
MOL007424	N/A	Artemisinin	15
MOL007426	N/A	Deoxyartemisinin	1

**Table 2:** The potential antihepatocarcinoma targets of *Artemisia annua*.

UniProt ID	Target protein	Target gene	Degree
P03372	6,8-Di-c-glucosylapigenin_qt oestrogen receptor	ESR1	22
P00533	Epidermal growth factor receptor	EGFR	22
P01106	Quercetin Myc protooncogene protein	MYC	21
P24385	G1/S-specific cyclin-D1	CCND1	21
P01133	Quercetin proepidermal growth factor	EGF	19
P35354	Quercetin prostaglandin G/H synthase 2	PTGS2	18
P08253	72 kDa type IV collagenase	MMP2	17
P14780	Matrix metalloproteinase-9	MMP9	17
P10275	Vitexin_qt androgen receptor	AR	17
P01100	Quercetin protooncogene c-Fos	FOS	16
P05121	Quercetin plasminogen activator inhibitor 1	SERPINE1	13
P08254	Quercetin stromelysin-1	MMP3	13
P11717	Quercetin insulin-like growth factor II	IGF2	12
P13500	Quercetin C-C motif chemokine 2	CCL2	12
P60568	Quercetin interleukin-2	IL2	11
P17936	Quercetin insulin-like growth factor-binding protein 3	IGFBP3	11
P08581	Hepatocyte growth factor receptor	MET	10
P04792	Quercetin heat shock protein beta-1	HSPB1	9
P08183	Multidrug resistance protein 1	ABCB1	9
P06400	Quercetin retinoblastoma-associated protein	RB1	8
P09211	Glutathione S-transferase P	GSTP1	7
Q01094	Quercetin transcription factor	E2F1	7
P04798	Quercetin cytochrome P450 1A1	CYP1A1	5
O15392	Quercetin baculoviral IAP repeat-containing protein 5	BIRC5	5

**Table 3:** Genes/signaling pathways related with hepatocarcinoma regulated by *Artemisia annua*.

GOID	Signaling pathway	Group genes
GO: 0042743	Hydrogen peroxide metabolic process	CCND1, CYP1A1, EGFR, ESR1, FOS, MMP3, PTGS2
GO: 0060749	Mammary gland alveolus development	AR, CCL2, CCND1, E2F1, EGF, EGFR, ESR1, GSTP1, IGFBP3, MYC
GO: 0071930	Negative regulation of transcription involved in G1/S transition of mitotic cell cycle	E2F1, RB1
GO: 2001028	Positive regulation of endothelial cell chemotaxis	HSPB1, MET
GO: 0045742	Positive regulation of epidermal growth factor receptor signaling pathway	EGF, MMP9
GO: 0032930	Positive regulation of superoxide anion generation	CRP, EGFR, GSTP1
GO: 1904705	Regulation of vascular smooth muscle cell proliferation	GSTP1, MMP2, MMP9

## Data Availability

The data used to support the findings of this study are available from the corresponding author upon request.

## Supplementary Materials

Supplementary Materials The characteristics of 19 compounds in PLP are shown in Supplementary Table 1.
