# Supplementary material for: A Network Pharmacology Approach to Reveal the Underlying Mechanisms of Artemisia annua on the Treatment of Hepatocellular Carcinoma
Source: Evid Based Complement Alternat Med. 2021 Feb 22;2021:8947304. doi: 10.1155/2021/8947304 (PMC7920725; doi:10.1155/2021/8947304)
Supplement: Supplementary Materials — The characteristics of 19 compounds in PLP are shown in Supplementary Table 1. [file 8947304.f1.pdf]

TABLE 1

Mol ID	Molecule Name	OB(%)	DL
MOL002235	EUPATIN	50.8	0.41
MOL000354	isorhamnetin	49.6	0.31
MOL000359	sitosterol	36.91	0.75
MOL004083	Tamarixetin	32.86	0.31
MOL004112	Patuletin	53.11	0.34
MOL000422	kaempferol	41.88	0.24
MOL000449	Stigmasterol	43.83	0.76
MOL004609	Areapillin	48.96	0.41
MOL005229	Artemetin	49.55	0.48
MOL000006	luteolin	36.16	0.25
MOL007274	Skrofulein	30.35	0.3
MOL007389	artemisitene	54.36	0.31
MOL007400	vicenin-2_qt	45.84	0.21
MOL007401	Cirsiliol	43.46	0.34
MOL007404	vitexin_qt	52.18	0.21
MOL007412	DMQT	42.6	0.37
MOL007415	[(2S)-2-[[(2S)-2-(benzoylamino)-3-phenylpropanoyl]amino]-3-phenylpropyl] acetate	58.02	0.52
MOL007423	6,8-di-c-glucosylapigenin_qt	59.85	0.21
MOL007424	artemisinin	49.88	0.31
MOL007425	dihydroartemisinin	50.75	0.3
MOL007426	deoxyartemisinin	54.47	0.26
MOL000098	quercetin	46.43	0.28
